# A New Mobile App to Train Attention Processes in People With Traumatic Brain Injury: Logical and Ecological Content Validation Study

**DOI:** 10.2196/64174

**Published:** 2025-04-09

**Authors:** Roxanne Laverdière, Philip L Jackson, Frédéric Banville

**Affiliations:** 1 School of Psychology Université Laval Quebec, QC Canada; 2 Department of Health Sciences Université du Québec à Rimouski Rimouski, QC Canada

**Keywords:** mobile app, attention training, cognitive remediation, mindfulness, psychometric properties, content validation

## Abstract

**Background:**

Attention is at the base of more complex cognitive processes, and its deficits can significantly impact safety and health. Attention can be impaired by neurodevelopmental and acquired disorders. One validated theoretical model to explain attention processes and their deficits is the hierarchical model of Sohlberg and Mateer. This model guides intervention development to improve attention following an acquired disorder. Another way to stimulate attention functions is to engage in the daily practice of mindfulness, a multicomponent concept that can be explained by the theoretical model of Baer and colleagues. Mobile apps offer great potential for practicing mindfulness daily as they can easily be used during daily routines, thus facilitating transfer. Laverdière and colleagues have developed such a mobile app called *Focusing*, which is aimed at attention training using mindfulness-inspired attentional exercises. However, this app has not been scientifically validated.

**Objective:**

This research aims to analyze the logical content validity and ecological content validity of the *Focusing* app.

**Methods:**

Logical content validation was performed by 7 experts in neuropsychology and mindfulness. Using an online questionnaire, they determined whether the content of the attention training app exercises is representative of selected constructs, namely the theoretical model of attention by Sohlberg and Mateer and the theoretical model of mindfulness by Baer and colleagues. A focus group was subsequently held with the experts to discuss items that did not reach consensus in order to change or remove them. Ecological content validation was performed with 10 healthy adults. Participants had to explore all sections of the app and assess the usability, relevance, satisfaction, quality, attractiveness, and cognitive load associated with each section of the app, using online questionnaires.

**Results:**

Logical content validation results demonstrated a high content validity index (CVI) of the attention training app. Excellent scores (CVI ≥0.78) in both the attention and mindfulness models were obtained for all exercises in the app, except 2 exercises. One of these exercises was subsequently modified to include expert feedback, and one was removed. Regarding ecological content validation, the results showed that workload, quality, user experience, satisfaction, and relevance of the app were adequate. The Mobile Application Rating Scale questionnaire showed an average quality rating between 3.75/5 (SD 0.41) (objective quality) and 3.65/5 (SD 0.36) (subjective quality), indicating acceptable quality. The mean global attractiveness rating from the AttrakDiff questionnaire was 2.36/3 (SD 0.57), which represents one of the strengths of the app.

**Conclusions:**

Logical and ecological content validation showed that *Focusing* is theoretically valid, with a high level of agreement among experts and healthy participants. This tool can be tested to train attention processes after a neurological insult such as traumatic brain injury.

## Introduction

### Attention Process Training

Attention is the ability to select relevant stimuli from the environment and inhibit those that are not relevant [1]. This cognitive process makes it possible for people to direct attention to the most salient elements to handle them appropriately. Indeed, attention is a factor of cognitive efficiency that supports memory, perception, and problem-solving [2]. Attention deficits are associated with significant difficulties in instrumental activities of daily living that influence the overall quality of life of individuals whose cognition is compromised [3,4].

Over the past decades, several experts have recommended the use of cognitive remediation, a nonpharmacological approach, for individuals with attention deficits [5].

People who live with attention deficits associated with a condition, such as traumatic brain injury (TBI) or attention-deficit/hyperactivity disorder, may benefit from attention training interventions [5]. This kind of intervention, particularly metacognitive strategy training (ie, anticipate, plan, seek, self-regulate, and evaluate), has been proven effective in neurological populations, such as people with TBI [6]. Cognitive remediation aims to improve cognitive functioning either by training to overcome deficits in functions or by allowing individuals to acquire strategies to best exploit their residual functions and help them engage in their daily activities [7]. Attention Process Training-III (APT-III) [8] is one of the most validated attention training programs for people with TBI. It includes two 45-minute sessions per week for 6 weeks. It includes two 45-minute sessions per week for 6 weeks, comprising visual and auditory exercises aimed at training the 5 attentional components of the model from Sohlberg and Mateer [9]. However, several of the studies addressing the effectiveness of this intervention have significant methodological limitations, including a lack of reliability and validity, and a low number of participants. Moreover, the results of the studies showed improvements among individuals in different attentional tests without any evidence of transfer to functional tasks [6].

According to the results of several studies [10], it is possible to stimulate and optimize attention functions using mindfulness practice. Indeed, mindfulness is a state of consciousness that emerges from deliberately focusing on an object in the present moment [11]. An analysis of the “cognitive mechanics” of mindfulness revealed that attention, working memory, and their different processes are key [1,10]. Indeed, Bishop et al [12] proposed 2 key components of mindfulness: self-regulation of attention (ie, the ability to maintain attention) and orientation to the present moment with curiosity, openness, and acceptance (ie, focusing on internal or external stimuli, inhibition, or activation of certain thoughts). Mindfulness interventions have shown promising results for different populations in terms of attentional capacities, particularly regarding sustained attention and attentional flexibility [13]. For instance, a previous study from our team [14] showed that a mindfulness intervention integrated into an ecological virtual environment was safe, feasible, and acceptable for people with mild TBI. The use of attentional focusing techniques of mindfulness could also be useful for the management of attention difficulties in people who have experienced moderate to severe TBI. Indeed, focusing on the present moment is a way of controlling one’s own attentional processes. As such, attention is considered central to the construct of mindfulness [10]. One limit of this type of intervention is access to a trained guide to accompany individuals, especially for people living in remote areas. It is therefore relevant to elaborate mindfulness interventions accompanied by a remote guide that is accessible from home, regardless of the region.

Although not everyone uses technology or is familiar with technology, mobile devices are ubiquitous in everyday life, and their apps are increasingly being used and perfected, particularly in health domains [15]. Countless mindfulness-based mobile apps have been developed; for instance, a 2021 systematic review identified 605 mindfulness-based mobile apps in European app stores [16]. Several meta-analyses confirmed the effectiveness of mindfulness interventions via mobile apps, particularly for mental health [17-19]. A study also showed that mindfulness interventions via a mobile app have effects comparable to mindfulness interventions in person [20]. These apps contain guided mindfulness exercises inspired by traditional approaches (mindfulness-based stress reduction [MBSR] [11] and mindfulness-based cognitive therapy [MBCT] [21]) that can be performed by individuals at home. However, even if studies show that these apps work, there is a lack of studies in the literature supporting the content and quality of these apps [22-25], and no studies have been conducted on the use of validated mindfulness apps in individuals with TBI. A systematic review in 2019 identified 53 mobile apps dedicated to people with TBI on the Apple App Store and Google Play Store [26]. Among these, there were compensatory measures, and some apps were aimed more at the remediation of cognitive functions, but none were aimed specifically at training attention using mindfulness. Findings from studies on the effectiveness of these apps in improving TBI symptoms are inconsistent. There is very limited research to validate the content of these apps, which poses a risk to users. Indeed, a specific validation is required to use these types of apps, considering that people who have experienced TBI (more specifically moderate or severe TBI) often have a variety of physical and cognitive deficits that can affect their ability to perform a mindfulness program via a mobile app. Physically, they may have motor disorders (paralysis, muscle weakness, and balance disorder), persistent fatigue, or sensory disorders (vision or hearing). Cognitively, deficits may involve attention, memory, language, and executive functions such as problem-solving and decision-making. These limitations may also be accompanied by emotional or behavioral disorders.

**Figure 1:**
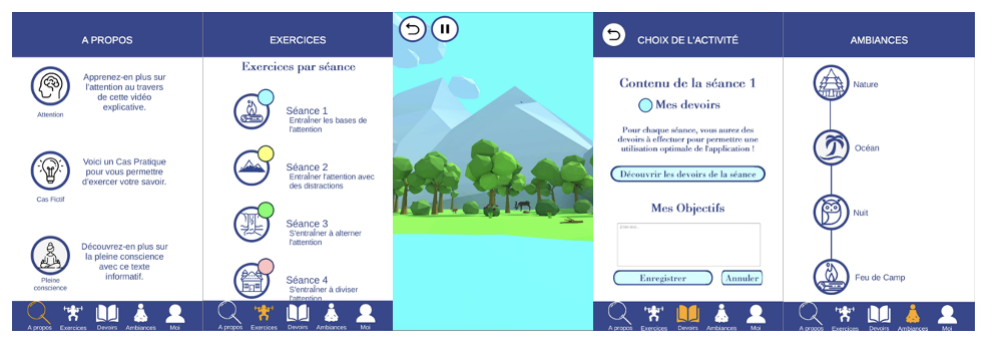
Screen captures of the different sections of the attention process training app (Focusing app) for people with traumatic brain injury.

To explore the potential of mobile apps in the training of attention via a mindfulness focusing technique, we developed an app, called *Focusing*, based on the Obesity-Related Behavioral Intervention Trials (ORBIT) intervention development model [27], which aimed at attention process training following the occurrence of neurological disorders such as TBI [28]. *Focusing* is currently available in French. The mobile app consists of mindfulness-inspired attentional focus exercises (Figure 1). The proposed attention training is based on 2 validated theoretical models: the mindfulness model of Baer et al [29] and the theoretical attention framework of Sohlberg and Mateer [9]. *Focusing* is composed of 5 sections (Multimedia Appendix 1). The first section “About” contains an interactive tutorial, a psychoeducational video about attention after TBI with a thought-provoking case example, and explanatory text about mindfulness. The second section “Exercises” contains the 4 attentional focus exercise sessions of the app. Each session includes 4 exercises (the first exercise is the same for each session to promote a state of calm conducive to the exercises of the session). Each session involves a component of attention, ranging from the simplest to the most complex. The third section “Homework” contains a list of homework assignments for each week. It also contains a “Goals” section, allowing the participant to set objectives and to consult with them throughout the week. The fourth section “Ambiences” contains ambient sounds representing nature and is aimed at freely practicing attentional focus in the visual and auditory modalities. The fifth section “Me” contains the participant’s profile.

### Theoretical Framework

The development of the attention training app was based on the neuropsychological model of attention of Sohlberg and Mateer [9]. This model was developed based on data from populations with brain damage and includes the subcomponents of attention that are frequently affected by TBI. This model has been widely used to explain attention in general, regardless of the neurological disorder. It involves the processes of focused, sustained, selective, alternating, and divided attention. *Focused attention* represents the ability to respond specifically and in a targeted way to a sensory stimulus. *Sustained attention* is the ability to maintain a consistent behavioral response during continuous or repetitive activity. *Selective attention* represents the ability to maintain a given behavioral or cognitive response to stimuli exerting a distracting or competitive effect. *Alternating attention* shifts the focus between tasks with distinct cognitive requirements efficiently and repeatedly. Finally, *divided attention* represents the ability to perform multiple tasks simultaneously.

To integrate this theoretical model into the *Focusing* app, each of the 4 sessions trains one of the components of the model by Sohlberg and Mateer [9]: Session 1, sustained attention; Session 2, selective attention; Session 3, alternating attention; and Session 4, divided attention. Focused attention, being a basic process, is trained in all sessions. The order of the sessions was determined to train the simplest forms of attention (sustained and selective) before the most complex forms (alternating and divided).

The development of the *Focusing* app was also based on the 5-facet model of mindfulness of Baer et al [29]. This model describes mindfulness as a multidimensional construct. It includes the following 5 components: observation, description, acting mindful, nonjudgment of inner experience, and nonreactivity to inner experience. *Observation* refers to noticing or paying attention to internal and external experiences, such as sensations, cognitions, emotions, images, sounds, and smells. *Description* refers to labeling internal experiments with words. *Acting mindful* includes being attentive to your current activities and may be opposed to behaving mechanically while one’s attention is focused elsewhere (often called autopilot). *Nonjudgment of inner experience* is about refraining from assessing thoughts and feelings. *Nonreactivity to inner experience* is about letting thoughts and feelings come and go, without being taken in or carried away by them.

To integrate the 5 mindfulness components of this model into *Focusing,* we selected and modified mindfulness exercises to fit the definitions of the components. The objective was for the intervention exercises to be broadly representative of all aspects of the model by Baer et al [29]. The components have therefore been integrated into the attentional exercises included in the 4 sessions.

### Objectives

This study aimed to assess the content validity of the *Focusing* app. This research, inspired by the previous work of Vogt et al [30], had the specific goal of analyzing the logical content validity and ecological content validity of the attention training program delivered through the mobile app. Logical content validation aims to test whether the app and the proposed exercises are aligned with “an operational definition” of the theoretical framework. More specifically, experts were instructed to decide on the level of proximity between the proposed exercises and the theoretical constructs presented in this study. Ecological content validation (on the basis of user experience in everyday life) aims to determine, before the proof-of-concept stage with a clinical population, the ease of use of the mobile app and the comprehensibility of the exercises (including vocabulary and visual/auditory aspects) that are integrated in the app.

## Methods

### Early Version and Preliminary Work

An initial app was developed based on meditation exercises from the MBSR [11] and MBCT [21] approaches. Mindfulness and neuropsychology experts (N=7) were subsequently consulted with a Delphi approach to obtain their views on the exercises. In general, the experts recommended using a mindfulness intervention approach in its entirety. Indeed, it is not recommended to develop an intervention that modifies or combines 2 approaches. The experts strongly suggested starting over by doing something completely different from existing mindfulness approaches. In response to the recommendations of the first group of experts, a new version of the *Focusing* app for training attentional functions was developed by Laverdière et al [28] and was subjected to validation in this study.

### Content Validation

Face validity represents the subjective feeling of the validity of an instrument [31,32]. It is not strictly about what the instrument measures, but what it seems to measure according to the research team. An instrument with good apparent validity will generate more positive attitudes, greater involvement in the task, and greater authenticity in the responses, which will increase the validity of the task performed [33]. For the development of the *Focusing* app, the research team implemented mindfulness-inspired attentional focus activities based on the understanding of the theoretical model of attention by Sohlberg and Mateer [9] and the model of mindfulness by Baer et al [29]. The construction of the intervention is based on the ORBIT model [28], which is considered valid by the research team.

Content validity assesses the extent to which the various items of an instrument are representative of the construct and its different facets [30,31]. Haynes et al [34] proposed 7 essential rules for validating the content of an instrument: Rule 1, rigorously define the domain and facets of the measured construct and validate this definition; Rule 2, use a sample of experts and members of the target population to create the items and define other aspects of the instrument; Rule 3, submit all aspects of the instrument to content validation; Rule 4, consult several experts to validate the content of the instrument and quantify their judgments using formalized scales; Rule 5, examine the proportional representation of items or tests relative to the different facets of the construct; Rule 6, present the results of content validation when publishing any new instrument; and Rule 7, consider all subsequent psychometric analyses to refine content validation.

To develop the *Focusing* app, validated theoretical models of attention and mindfulness were used (Rule 1) [9,29]. Mindfulness and attention training experts were first consulted when the intervention was created (Rule 2). Moreover, the entire attention process training was validated by experts and people from the general population (Rules 2, 3, and 4). The validation data allowed to refine the intervention and thus to have a development procedure and a valid intervention (Rule 5). Studies on the validation process are presented in this manuscript (Rule 6). All the data obtained will make it possible to reflect comprehensively on the modifications to be made to the app for improvement before testing the app in a clinical population (Rule 7).

### Ethical Considerations

Ethics approval was obtained for this study from the *Centre Intégré Universitaire de Santé et de Services Sociaux de la Capitale-Nationale* (CIUSSS-CN) rehabilitation and social integration committee (approval number: MP-13-2022-2523). Written consent was obtained from all participants after they were informed about the study’s objectives and potential benefits. Participation was completely voluntary, and participants retained the right to withdraw at any moment without prejudice. To protect their privacy, all data were kept strictly confidential and anonymized, ensuring that participants could not be identified. No financial compensation or incentives were offered for participation.

## Study 1: Logical Content Validation

### Participants

Logical content validation was conducted by a panel of 7 experts from Canada (n=5) and France (n=2) who participated in the validation process in February 2023. The selected experts had to meet the following inclusion criteria: (1) be a clinician, have at least 5 years of clinical experience with moderate or severe TBI clients, or have at least 5 years of experience in the mindfulness practice; (2) be a neuropsychologist, psychologist, or occupational therapist; and (3) have practiced or be familiar with physical rehabilitation. The experts were recruited via email from September 2022 to January 2023. A total of 21 experts were contacted. Of these 21 experts, 10 agreed to participate; however, 1 expert withdrew and 2 did not complete all the validation steps. The participants included 4 clinical neuropsychologists involved in teaching and research. They specialize in the development of techniques for evaluation or intervention in different clinical populations (TBI, autism spectrum disorder, and neurodegenerative diseases). The other 3 participants are clinical psychologists involved in teaching and research. They specialize in mindfulness interventions in pediatric and adult populations.

### Procedure

Experts who agreed to participate in the study received an email containing information about the exercises and a link to download a desktop version of the *Focusing* app, which they were asked to explore over a 4-week period. The desktop version of the app corresponds to a preliminary version of the implementation on the Google Play Store and is visually identical to the currently available app on Android. The experts were invited to try the different sections of the app and to perform all the exercises of the program. They were asked to assess whether the exercises in the program are theoretically valid according to the theoretical model of attention by Sohlberg and Mateer [9] and the theoretical model of mindfulness by Baer et al [29], using a questionnaire on the Lime Survey platform (closed survey). In the questionnaire, experts had access to the definitions of the attentional components of the model by Sohlberg and Mateer [9] and the 5 components of the model of mindfulness by Baer et al [29] because their respective knowledge of the theoretical models was different. In addition, the component of attention trained in each exercise was identified. The experts had to assess whether each exercise involves the targeted component of attention, using a Likert scale ranging from 1 (totally disagree) to 4 (totally agree). Using the same Likert scale, they also had to assess the extent of training of each of the 5 components of mindfulness, in general, in the exercises. Indeed, the 5 components need not be present in all the exercises, but rather they should be in the mindfulness approach used for the construction of the exercises. They also had to comment on the exercises and suggest improvements when they considered that an item was not theoretically valid. Finally, the experts had to assess the relevance of the first exercise of each session, using a Likert scale ranging from 1 (totally irrelevant) to 4 (totally relevant). The questionnaire consisted of 35 items and 20 pages. The experts could see their progress in the questionnaire using a progress bar at the top of the page. They could also save their answers and resume the questionnaire later using their email and a password. They also had the option to change their answers and go back in the questionnaire. No cookies were used. The experts’ responses were anonymous. Data and comments were then analyzed, and a discussion meeting with the experts (videoconference) was subsequently held to discuss the elements that did not reach consensus between the experts. The changes suggested by the experts were then applied to the mobile app. The experts concluded this meeting in a consensual manner by saying that they would consider the app to be valid once their suggestions were incorporated. After making the adjustments and incorporating the experts’ suggestions, one of the experts reviewed the entire content of the intervention to validate it again. This expert specializes in adapting mindfulness interventions to different clinical populations. In addition, one of the mindfulness experts trained the person who lent her voice when recording voice tone and speed exercises. Once this step was completed, the usability of the new version of the mobile app was tested.

### Data Analysis

For the evaluation of the content of the app and the theoretical model of Sohlberg and Mateer [9] regarding attention and that of Baer et al [29] regarding mindfulness by experts, a Likert-type scale was used with 4 possible responses: 1, I totally disagree; 2, I somewhat disagree; 3, I somewhat agree; and 4, I totally agree. For this analysis, the content validity index (CVI) was calculated from the count of the responses “I somewhat agree” and “I totally agree” divided by the total number of experts. According to the literature, the items evaluated must have a CVI greater than or equal to 0.78 (78%); otherwise, they need to be adjusted according to experts’ suggestions [35].

### Results

The experts assessed the theoretical validity of each exercise of the sessions in relation to the attentional model used [9] for a total of 16 exercises. They also assessed the theoretical validity of the attention training program in relation to the mindfulness model used [29].

Regarding the validity of the attention model (Table 1), the calculated CVI for 14 exercises (out of 16) ranged between 0.86 and 1.00, indicating that these exercises are valid [35]. One exercise with a CVI value of 0.71 required revision. The experts mentioned that this exercise was too focused on mindfulness concepts, which strayed away from the concept of selective attention. They also pointed out that more distractions should be present in the exercise. In addition, experts considered another exercise (Exercise 4.4) to be not valid, as it had a CVI value of 0.42, and thus, it was eliminated. According to the experts, Exercise 4.4, which consisted of a dual visual-auditory task, was too demanding for people with acquired brain injury. As mentioned above, the first exercise of each session is the same to allow participants to get into a state conducive to exercises aimed at training the different components of attention. The relevance of repeating this exercise was assessed by the experts. The calculated CVI was 0.86, indicating that the repetition of this exercise is relevant to the program.

For validation related to the mindfulness theoretical framework (Table 2), the calculated CVI for all 5 components of the model ranged between 0.86 and 1.00, indicating that the remediation program is valid with regard to its mindfulness component.

**Table 1:** Content validity index and data of each exercise based on the attention model by Sohlberg and Mateer [9], as evaluated by expert judges.

Instrument item^a^		CVI^b^	Mean (SD)	Modification
**Session 1: focused and sustained attention**				
	Exercise 1.1	0.86	3.33 (0.47)	Minor
	Exercise 1.2	0.86	3.29 (0.70)	Minor
	Exercise 1.3	0.86	3.29 (0.70)	Minor
	Exercise 1.4	0.86	3.14 (0.64)	Minor
**Session 2: selective attention**				
	Exercise 2.2	0.71^c^	3.43 (0.90)	Major
	Exercise 2.3	0.86	3.29 (0.70)	Minor
	Exercise 2.4	0.86	3.14 (0.64)	Minor
**Session 3: alternating attention**				
	Exercise 3.2	0.86	3.29 (0.70)	Minor
	Exercise 3.3	1.00	3.71 (0.45)	No change
	Exercise 3.4	0.86	3.14 (0.64)	Minor
**Session 4: divided attention**				
	Exercise 4.2	0.86	3.57 (0.73)	Minor
	Exercise 4.3	0.86	3.57 (0.73)	Minor
	Exercise 4.4	0.42^c^	2.71 (0.88)	Item deleted

^a^Exercises 2.1, 3.1, and 4.1 are not included in the table as they have the same content as Exercise 1.1.

^b^CVI: content validity index.

^c^Items with a CVI below the acceptable level of 0.78.

**Table 2:** Content validity index and data of each component of the theoretical framework of mindfulness by Baer et al [29] for the whole training according to expert judges.

Instrument item	CVI^a^	Mean (SD)	Modification
Observing	1.00	3.86 (0.35)	No change
Describing	0.86	3.00 (0.53)	Minor
Acting with awareness	0.86	3.57 (0.73)	Minor
Nonjudging of inner experience	1.00	3.71 (0.45)	No change
Nonreactivity to inner experience	1.00	3.57 (0.49)	No change

^a^CVI: content validity index.

#### Synthesis of Expert Recommendations

During the logical content validation, 4 dimensions were explored by the experts: theoretical model of attention, theoretical model of mindfulness, technological aspects, and audio. Regarding the theoretical model of attention, the recommendations of the experts were to: (1) increase the duration of exercise in sustained attention; (2) increase distractions in selective attention; and (3) remind more frequently about the instructions in all exercises. Regarding the theoretical model of mindfulness, the recommendations of the experts were to: (1) simplify the vocabulary; (2) give more concrete examples to the participants; and (3) decrease the speed of the voice in the audio tracks of the exercises. In terms of technology, the experts reported some inconsistencies between the visual elements of the app and the audio content of the exercises.

Exercises were modified to reflect these recommendations. First, the sustained attention exercises (1.1 to 1.4) were extended by a few minutes each. Visual and auditory distractions were added in the selective attention exercises (2.1 to 2.4). Throughout the training exercises, the speech was adapted by adding reminders of instructions several times during a single exercise. The vocabulary was also adapted to be accessible to the general population, for example, by adding concrete examples and adapting the tone of voice (speaking less quickly). The technological inconsistencies raised by the experts were also corrected.

## Study 2: Ecological Content Validation

### Participants

For this study, a convenience sample of 10 healthy participants (7 female and 3 male participants) was recruited from the social networks of the research laboratory. Participants were required to meet the following criteria: (1) be over 18 years of age; (2) understand and speak French; (3) own an Android device; (4) have no diagnosis of TBI; (5) have no serious mental disorder including but not restricted to schizophrenia spectrum disorder, bipolar disorder, intellectual disability, and attention deficit disorder; (6) have no vestibular disorder; and (7) have no substance abuse.

An academic researcher also explored the mobile app. This expert is interested in the neuropsychology of rehabilitation, particularly among adults who have experienced TBI or stroke. This researcher’s work also focuses on the use of technology in clinical neuropsychology (mobile devices, teleneuropsychology, and virtual reality). This expert did not complete the validation questionnaires, but this expert’s comments were taken into consideration in the validation process.

### Procedure

The 10 participants completed the entire study remotely. Interested participants were directed to an online eligibility questionnaire via a link in the recruitment advertisement. Eligible participants received the mobile app download procedure. They then had 2 weeks to explore by themselves the mobile app and fill out the questionnaires provided for the experimental protocol via Lime Survey (open survey). The self-administered questionnaires were: a sociodemographic questionnaire, the Simulation Task Load Index (SIM-TLX) questionnaire (mental load; [36]), the French version of the Mobile Application Rating Scale (MARS-F) questionnaire (quality of the app; [37,38]), the French version of the AttrakDiff questionnaire (user experience; [39,40]), and a questionnaire concerning the participants’ satisfaction with the elements of the app as well as their perception of the relevance of these elements. The whole questionnaire set consisted of 107 items and 9 pages. Participants could see their progress in the questionnaire on a progress bar at the top of the page. They could also save their answers and resume the questionnaire later using their email and a password. They also had the option to change their answers and go back in the questionnaire. No cookies were used. The participants’ responses were anonymous. Once all participants completed the exploration, questionnaire responses were analyzed and changes to the mobile app were made if problematic elements were identified.

### Instruments

#### SIM-TLX Questionnaire

This questionnaire [36] provides a measure of participants’ cognitive load. It contains 9 scales (mental demands, physical demands, temporal demands, frustration, task complexity, situational stress, distractions, perceptual strain, and task control). The responses range from 1 (very low) to 20 (very high).

#### MARS-F Questionnaire

This questionnaire [37,38] provides a measure of the app’s quality. Items are rated on a scale of 1 to 5 (1=inadequate; 2=poor; 3=acceptable; 4=good; and 5=excellent). The MARS-F questionnaire comprises 4 main scales: (1) user engagement (5 items: entertainment, interest, customization, interactivity, and target group); (2) functionality (4 items: performance, usability, navigation, and gestural design); (3) esthetics (3 items: layout, graphics, and visual appeal); and (4) information quality (7 items: accuracy of app description, goals, quality of information, quantity of information, quality of visual information, credibility, and evidence base). For each scale, a mean score was calculated.

#### AttrakDiff Questionnaire

This questionnaire [39,40] provides a measure of the app user experience. It provides data on 3 quality scales (pragmatic, hedonic-stimulation, and hedonic-identity) and a scale of global attractiveness. Each item is rated on a scale ranging from –3 to +3. Values close to the average (between –1 and 1) are standard; they mean that the product meets its objective. Values outside this neutral zone are to be considered as positive (from 1 to 3) or negative (from –1 to –3) points of the app.

#### Satisfaction Questionnaire

In this questionnaire, participants rated on a scale of 1 (totally dissatisfied) to 5 (totally satisfied) their levels of satisfaction regarding the 5 sections (About, Exercises, Homework, Ambiences, and Me) and the functionalities of the app. Participants also rated the relevance of each section and functionality (technological issues, sound, and ability to navigate) on a scale from 1 (totally irrelevant) to 5 (totally relevant). They also documented the technical issues they encountered while exploring the app.

### Data Analysis

To compute for all participants the variables surveyed by the questionnaires, central trend measurements (mean and SD) and frequency analyses were performed on the data. Exploratory correlations were made on the data.

### Results

A total of 11 participants started the usability validation study. Only 10 participants (7 female and 3 male participants) completed the validation process of the app. Their mean age was 51 (SD 15) years, and the mean number of years of education was 13 (SD 4). Most participants (5/10, 50%) worked full-time, 40% (4/10) were retired, and 10% (1/10) were studying full-time. None worked in the technology industry. Moreover, 80% (8/10) of the participants regularly watched animated films and only 40% (4/10) regularly played video games. The smartphone was the most used technological tool by participants (9/10, 90% used it daily) followed by computers (4/10, 40% used it daily) and tablets (2/10, 20% used it daily). On a scale of 1 to 10, participants’ comfort levels with these technological tools averaged 7.36 (SD 1.57) for smartphones, 5.55 (SD 2.58) for computers, and 5.45 (SD 2.62) for tablets.

The results of the different questionnaires are presented in Table 3.

**Table 3:** Scores of the scales of the questionnaires for usability validation of attention process training according to healthy participants.

Questionnaire scale			Score, mean (SD)		Range, minimum to maximum		
**SIM-TLX^a^ (range: 1 [very low] to 20 [very high])**							
	Mental demands	4.73 (2.33)		1 to 8			
	Physical demands	1.45 (1.04)		1 to 4			
	Temporal demands	1.36 (0.81)		1 to 3			
	Frustration	1.45 (1.21)		1 to 5			
	Task complexity	3.82 (2.27)		1 to 7			
	Situational stress	1.18 (0.60)		1 to 3			
	Distractions	5.55 (3.59)		1 to 11			
	Perceptual strain	2.64 (4.20)		1 to 15			
	Task control	2.73 (2.41)		1 to 8			
**MARS-F^b^ (range: 1 [low quality] to 5 [high quality])**							
	Engagement	3.98 (0.48)		1 to 5			
	Functionality	4.30 (0.45)		3 to 5			
	Esthetic	3.80 (0.45)		3 to 5			
	Information	2.89 (0.60)		1 to 5			
	Objective quality	3.74 (0.41)		1 to 5			
	Subjective quality	3.65 (0.36)		2 to 5			
**AttrakDiff (range: –3 to 3)**							
	Pragmatic quality	1.94 (0.61)		0 to 3			
	Hedonic quality: Simulation	1.79 (0.55)		–2 to 3			
	Hedonic quality: Identification	1.51 (0.57)		–2 to 3			
	Global attractivity	2.36 (0.57)		0 to 3			
**Satisfaction (range: 1 [totally dissatisfied/irrelevant] to 5 [totally satisfied/relevant])**							
	Satisfaction	4.30 (0.62)		2 to 5			
	Relevance	4.41 (0.47)		2 to 5			

^a^SIM-TLX: Simulation Task Load Index.

^b^MARS-F: Mobile Application Rating Scale, French version.

The results showed that the app requires low physical and temporal demands according to the SIM-TLX questionnaire. All items scored less than 4. Even if the overall results on the 9 scales were low, mental demand, distractions, and task complexity were rated slightly higher than the others. The app generated a low level of frustration and stress (<1.5). Participants had a good sense of control when using the app (a small score indicates a good sense of control).

The results of the MARS-F questionnaire showed that, in general, the quality of the app was adequate according to the participants. The average quality rating for the app was between 3.75/5 (SD 0.41) (objective quality) and 3.65/5 (SD 0.36) (subjective quality), demonstrating moderate quality [37,38]. The results indicated that the app is functional and esthetic and provides an adequate level of engagement. The information scale had a lower score (mean 2.89, SD 0.60). This suggests that participants did not have enough information about the app development process to judge its credibility.

The result of AttrakDiff’s global attractiveness scale indicated that the user experience was adequate, with a mean score of 2.36 (SD 0.57). The result of the pragmatic quality scale, which describes the usability of the app, indicated that using the app was easy. The result of the hedonic quality scale-stimulation indicated that the app appeared new, interesting, and stimulating. The result of the hedonic quality scale-identity indicated that participants liked to interact with the app (eg, voice). In fact, all results were between 1 and 3 on the –3 to +3 scale, which points out the positive aspects of the app.

The satisfaction questionnaire showed that participants were generally satisfied with the different sections and functionalities of the app. They also considered that the different sections and functionalities of the app were relevant.

#### Synthesis of Correlation Analyses

A positive and strong correlation was obtained between the participants’ ease of using a tablet and their ease of using a phone (*r*=0.832; *P*=.001). This suggests that *Focusing* could be used on a tablet as well.

A negative and moderate correlation was obtained between the result of the cognitive load of participants (SIM-TLX) and the functionality score of the app (*r*=–0.635; *P*=.049). Better functionality was therefore associated with a smaller mental load. Having obtained a high score in functionality (MARS-F), the app does not appear to lead to mental overload that could interfere with the attention processes of participants.

A positive and moderate correlation was obtained between pragmatics (ease of use; AttrakDiff) and engagement level (MARS-F) (*r*=0.657; *P*=.04). The ease of use of *Focusing* therefore increases participants’ commitment to the intervention. Better engagement was positively and strongly correlated with the subjective quality (MARS-F) of the app perceived by the participant (*r*=0.821; *P*=004). The level of satisfaction of participants with the different sections of *Focusing* was also positively and moderately correlated with objective quality (MARS-F) (*r*=0.669; *P*=.04) and positively and strongly correlated with the overall level of attractiveness (AttrakDiff) of the app (*r*=0.919; *P*=.001).

## Discussion

### Main Results

Despite the growing popularity of mobile apps for cognitive training and mindfulness, there is still a lack of comprehensive analysis supporting the content and quality of these apps [22-25]. A systematic review identified 53 commercial cognitive training or rehabilitation apps in 2023 [23]. Among these apps, only 5 had an overall MARS score exceeding 4 points, and more than half had a score of less than 3 points. Considering that a MARS score of 3 points is acceptable, most of these apps did not even meet the acceptable criteria for quality [37,38]. Furthermore, to our knowledge, none of these apps were intended to address attention in people who have moderate or severe TBI.

Furthermore, no studies on the development of attention interventions identified in the literature used a validated development model. Due to the existing gaps in the methodology of studies on attention remediation interventions, it is essential to use a model like ORBIT in the development of future interventions to address these limitations. Promising behavioral interventions in development are often abandoned rather than refined or improved if they fail the initial tests. In some cases, successful interventions in early studies are not pushed toward more rigorous efficacy studies, while others are tested in efficacy studies prematurely.

This research aimed to validate the content and user experience of an attention training intervention developed using the ORBIT model via a mobile app called *Focusing*. The first step was to validate the logical content with experts to ensure that the exercises train the attention components of the model by Sohlberg and Mateer [9]. It also aimed to validate that the remediation program sessions incorporate the 5 components of the mindfulness model by Baer et al [29]. The results showed that experts (including experts in cognitive functions and experts in mindfulness) considered the intervention to be theoretically valid according to the 2 models. Regarding the theoretical model of attention, the experts validated that each session of the intervention involves a component of the attentional model. Following the expert logical validation process, 1 exercise was removed, 1 underwent major revisions, 10 were improved according to the recommendations, and 1 was not changed. The removed exercise, which consisted of a dual visual and auditory task, was deemed too demanding for people with acquired brain injury. Major changes included increasing the duration of exercise, increasing the level of distraction, and making the instructions more clear. Moreover, the experts confirmed that the 5 components of the mindfulness model are included throughout the exercises. Changes were made to the exercises as recommended by the experts: the vocabulary for the instructions was simplified, concrete and more practical examples were added, the speed of the voice track/instructions was reduced, and pauses were added to the voice track.

The second step was the ecological content validation of the *Focusing* app. The relevance, satisfaction, quality, attractiveness, and workload associated with each section of the app were evaluated by participants from the general population.

The results of the SIM-TLX questionnaire underlined the level of workload when using the app, which involves low physical and temporal demands. It also involves low stress and frustration. These results confirm that the *Focusing* app respects the principles of mindfulness. Indeed, mindfulness promotes being in the present moment and the acceptance of the experience. The scales of the SIM-TLX questionnaire associated with higher workload are mental demand, distraction, and task complexity. The results confirm that attentional training program exercises require some cognitive effort. In addition, distractors were deliberately integrated into the exercises to train selective attention.

MARS is one of the most widely used questionnaires in the literature for app quality assessment [37]. This questionnaire was also used by clinicians to assess the quality of 23 mindfulness apps available on the Apple App Store and Google Play Store [25]. The results showed that the app is of good quality and is esthetic, functional, and engaging. These features allow you to properly interact with the app. Resources can therefore be entirely devoted to the training of attention. The Quality Information scale had the worst result. To prevent misinformation and adverse effects from *Focusing* app use, information quality must be improved. In fact, wrong or misleading information could result in a decrease in user safety [41]. Informing participants of the involvement of experts in the development process (eg, psychotherapists and researchers in mindfulness or cognition training) might help to address this problem. Moreover, a better description of the app on the Play Store might improve information quality.

The results of the AttrakDiff questionnaire demonstrated good user experience of the *Focusing* app. The app was easy to use, interesting, and challenging. It was also pleasant to interact with the app. This also means that the voice (tone and speed) used for the exercises was adequate. The results showed that the recommendations of the experts during the logical content validation helped improve the user experience of the app.

In comparison with our study, the attention training application (ATA) developed by Hill et al [42,43] is an app aimed at training the attention of elderly people without cognitive impairment. To our knowledge, this app is the most recent in the literature to be validated with the target population. The ATA is an adaptation of the «dual n-back» computerized intervention [44]. This study did not use a validated intervention development model such as ORBIT. The app was, however, validated twice [42,43] with a small number of elderly people and using a mixed methods study design. This study corresponds to Phase I(b)-Refine of the ORBIT model. As in our study, participants were asked to complete questionnaires to capture familiarity with technology and to provide their general impressions of the ATA on a scale from 0 (negative) to 10 (positive in 4 categories: overall, easy, satisfying, and interesting). The User Interface Satisfaction questionnaire was also used [45]. Feedback had to be given on a 7-point Likert-type scale for different items. Interviews were conducted to allow participants to share their perceptions of the app. A limitation of this previous study is that the quality of the app was not assessed with the MARS questionnaire. Moreover, the cognitive load associated with using the app was not assessed.

### Limitations

The content validation of the attention remediation program has some limitations. Although the logical content validation experts concluded that the program would be fully valid once their recommendations were incorporated, the revised program was only reviewed by 1 of these experts after changes were implemented. In addition, 1 of the experts could not be present at the focus group to discuss the recommendations.

The current validation of user experience was limited by the characteristics of the participants. First, the sample included more women (70%) than men (30%). However, only one-third of people with TBI in Canada are women [46,47]. In addition, most participants were in their 50s or 60s, while youth and young adults aged 12 to 19 years are 5.2 times more likely to have TBI [46]. Finally, participants in the sample considered themselves weakly to moderately comfortable with the use of electronic devices (computers, tablets, and smartphones), which may be related to the age of the participants. On the other hand, young people are more comfortable with the use of apps on smartphones. Different data about user experience could have been obtained with a sample that is very comfortable with technologies. However, by targeting people who are less skilled with technology, we ensured that this would not be an obstacle.

### Future Research

Future studies should assess the safety, tolerability, and acceptability of this attention training intervention via a mobile app in a population with moderate or severe TBI. More specifically, as part of phases Ib (refine) and IIa (proof-of-concept) of the ORBIT model [27], 5 to 10 people with moderate or severe TBI should be recruited as part of a multiple single-case experimental design. The test phase should include a waiting period and an intervention period, both marked by pretests and posttests. At the end of this phase, it will be possible to determine whether the intervention is clinically acceptable and whether it can be made available to a larger sample, leading to phase III (efficacy) studies that will involve a randomized controlled design. At the end of phase III, we also want to translate the tool for the English-speaking population in order to increase the recruitment pool. This will involve translating the audio files and the menus of the different sections of the app. We also want to develop an iOS version of the app to make it more accessible and expand recruitment.

### Conclusions

The *Focusing* app is one of the first mobile attention training apps scientifically validated with the ORBIT model and developed based on theoretical models of both attention and mindfulness. A systematic validation approach was used to ensure that the intervention was theoretically valid before clinical validation. In addition, this project provides a better understanding of the attentional processes modulated by attentional focus techniques based on mindfulness. In fact, this study attempts to dissect attentional processes from the angle of a theoretical model recognized in neuropsychology.

## Appendix

Multimedia Appendix 1 Description of the attention training exercises in each session and the attention components and mindfulness components trained in the exercises.

## Abbreviations

ATA: attention training application
CVI: content validity index
MARS-F: Mobile Application Rating Scale, French version
MBCT: mindfulness-based cognitive therapy
MBSR: mindfulness-based stress reduction
ORBIT: Obesity-Related Behavioral Intervention Trials
SIM-TLX: Simulation Task Load Index
TBI: traumatic brain injury

## Data Availability

The datasets generated and analyzed during this study are available from the corresponding author upon reasonable request.
