# Supplementary material for: A New Mobile App to Train Attention Processes in People With Traumatic Brain Injury: Logical and Ecological Content Validation Study
Source: JMIR Form Res. 2025 Apr 9;9:e64174. doi: 10.2196/64174 (PMC12018855; doi:10.2196/64174)
Supplement: Multimedia Appendix 1 [file formative_v9i1e64174_app1.docx]

| Session number | Attention components | Mindfulness components | Exercises |
| --- | --- | --- | --- |
| 1 | Focused and sustained | Observation Act  Non-judgment Non-reactivity | - 1. Breathing space: this exercise is repeated at the beginning of each session to promote connection in the here and now. Participants are around a fire and should focus on their breathing.   2. Breath meditation: participants are around a fire and focus is on breathing only. During the exercise, there is no sound.   3. See without naming: without sound, participants must pay attention to animated stimuli (campfire flames).   4. Hear without naming: without picture, participants should pay attention to the crackling of the campfire. |
| 2 | Selective | Observation Act  Non-judgment Non-reactivity | - 1. Breathing space.   2. Mountain meditation: the participant should focus only on the mountain, ignoring birds and animals. During exercise, he can hear the nature and animals/birds.   3. See without naming (with hearing distraction): there are different animals and birds at the foot of the mountain that make a course, the participant must pay attention only to the fox. During the exercise, the participant can hear the sounds of birds and nature.   4. Hear without naming (with visual distraction): the participant hears the sounds of birds and nature. He should focus on the bird sounds only. During the exercise, the participant can see the mountain scene with animals and birds. |
| 3 | Alterned | Observation Act  Description  Non-judgment Non-reactivity | - 1. Breathing space.   2. Exercise SIM card: participants are in front of the waterfall. They must focus their attention alternately on their sensations, thoughts and senses.   3. Leaf exercise: participants are in front of the waterfall. They should be aware of tree leaves that are sliding on the water. When they see green leaves, they must name a thought. When they see yellow leaves, they must name a sensation. In total, they will have to name 3 thoughts and 3 sensations.   4. See and hear without naming (alternating according to a 10-30 second sound index): See = participants should focus on the waterfall, Name = participants should focus on the water’s sound. Throughout the exercise, visual and sound are present. |
| 4 | Divided | Observation Act  Description  Non-judgment Non-reactivity | - 1. Breathing space.   2. Listening exercise: participants listen to a narrative text that comes from the cottage radio and focuses on mindfulness in order to summarize its content, while counting the number of times the word group “mindfulness” is said.   3. Visual exercise: participants read a newspaper on the cottage table that contains an educational text about attention, with the aim of summarizing its content, while counting the number of times the word “attention” appears in the text. |

**Multimedia Appendix 1.** Description of the attention training exercises in each session and the attention components and mindfulness components trained in the exercises.
